# Supplementary material for: Trends of tuberculosis case detection, mortality and co-infection with HIV in Ghana: A retrospective cohort study
Source: PLoS One. 2020 Jun 24;15(6):e0234878. doi: 10.1371/journal.pone.0234878 (PMC7313972; doi:10.1371/journal.pone.0234878)
Supplement: Attachment — Submitted filename: Response to reviewers PONE-D-20-02837R1.docx [file pone.0234878.s002.docx]

**RESPONSE TO REVIEWERS’ COMMENTS - MANUSCRIPT PONE-D-20-02837**

We are pleased to return the revised version of our manuscript to you for consideration. Please, find below the specific responses to the specific comments made by the reviewers. We have provided a step-by-step response to each reviewer’s comments. We have made track changes to all comments in the revised manuscript. Thank you for the opportunity to work with you on this paper and more papers to come.

**Editor’s comments**

**In your revised cover letter, please address the following prompts:**

a) If there are ethical or legal restrictions on sharing a de-identified data set, please explain them in detail (e.g., data contain potentially identifying or sensitive patient information) and who has imposed them (e.g., an ethics committee). Please also provide contact information for a data access committee, ethics committee, or other institutional body to which data requests may be sent.

**Response:** *There are no ethical or legal restrictions on data used in this study. The data has therefore been uploaded as a supporting Information file in the revision.*

b) If there are no restrictions, please upload the minimal anonymized data set necessary to replicate your study findings as either Supporting Information files or to a stable, public repository and provide us with the relevant URLs, DOIs, or accession numbers. Please.

**Response:** *Anonymized data set used for this study* *has been uploaded as Supporting Information file.*

4. Please amend either the title on the online submission form (via Edit Submission) or the title in the manuscript so that they are identical.

**Response:** *We have amended the title in the revised manuscript and have made it identical with the one on the online submission form.*

5. Your ethics statement must appear in the Methods section of your manuscript. If your ethics statement is written in any section besides the Methods, please move it to the Methods section and delete it from any other section. Please also ensure that your ethics statement is included in your manuscript, as the ethics section of your online submission will not be published alongside your manuscript.

**Response**: *Ethics statement was included in the Methods section. We have however, removed ethics statement from any other section in the revised manuscript (line 38).*

**Review Comments to the Author**

Reviewer #1:

Major points
- I was concerned about the link between smear positive disease and lowered death rate vs. smear negative disease. It is mentioned in the discussion with some reasonable explanations but could you also add in whether this has been seen in other settings?

**Response:** *Studies from different settings have reported different findings regarding the difference between sputum smear positive and smear negative TB patients and TB death. Alobu et al. for instance found that smear negative cases had 2.4 times increased odds of death compared to smear positive cases, while Carlos and others did not find any difference in their systematic review. We have discussed this further in the revised manuscript (line 377).*

***Minor points***
- you mention in the introduction that no one has looked at trends in CDR / mortality in Ghana - what about the WHO? Perhaps no one has looked at the lower district level that you are able to?

**Response:** *We have made the statement specific to the district level in the revised manuscript (line 102 of revised manuscript).*

- why did you use a different time period for treatment vs. mortality rate predictions? what impact might this have on your results?

**Response:** *Data used for assessing TB treatment outcomes are usually that of the previous year since some cases registered in the current year might still be on treatment in the current year because treatment last for at least 6 months. Therefore, we used 2013-2017 data to estimate CDR and used 2012-2016 to estimate mortality rates and predictors. This is global standard of reporting TB indicators.*

- how often was the data extraction systematically checked?

**Response:** *After extracting each year’s data, the district TB focal person was made to verify and compare the extracted data with the primary source.* *Discrepancies were checked and corrected accordingly. We have clarified this in the revised manuscript (line 145).*

-what is the p-value in Table 2 - univariate?

**Response:** *The p-values in Table 2 are from Chi-square test of significant difference between males and females with respect to the categorical variables.*

- table 3 - how was the 95% CI generated?

**Response:** *The 95% CI was generated using immediate command for variable distributed as binomial in Stata. TB cases tested for HIV were the observation and HIV positive patients were success. For more clarification visit ( http://www.stata.com/manuals13/rci.pdf )*

- bottom of the paragraph entitled: TB case fatality rates and trends, it should be "showed irregular trends" not "shown irregular trends".

**Response:** *We have corrected that as suggested.*

- Table 5 - is this the multivariate analysis? did you just combine all factors into one regression?

**Response:** *The table shows the results of both simple and multiple logistic regression. We have reported crude odds ratios (COR) for the simple regression analysis and adjusted odds ratio (AOR) for the multiple regression analysis in the table. Only variables that were statistically significant at p<0.05 in simple analysis were included in the multiple analysis but not all factors.*

- Discussion: I'm not sure that you want to say, "confirmed by Obasanya", rather that this was "also found to be the case" in Nigeria.

**Response:** *We meant that Obasanya and colleagues also found similar relationship. We have made this clear in the revised manuscript (line 275).*

- Discussion: replace "antibiotic microbial" resistance with just "antibiotic" resistance

**Response:** *Antibiotic microbial resistance has been replaced with antibiotic resistance.*

- Figures: expand CDR and in general the descriptions of what is shown

- Figure 1: label the target - Ghana's / WHO's / Global TB?

**Response:** *The target is the global target set by the end TB strategy of WHO. We have made it clear in the revised manuscript.*

- Figure 1: what is the blue line?

**Response:** *The blue line represents the study’s overall CDR. We have indicated that on the line in the figure in the revised manuscript.*

- Figure 2: what are the numbers? mean?

**Response:** *The numbers are the CDRs for each year.*

- Figure 3: please take the descriptions of what the colours mean from FIgure 4 and add to Figure 3

**Response:** *The colour meaning has been taken from Figure 4 and added to Figure 3.*

- Figure 4: what happened in Ketu South in 2012?

**Response:** *Response: There were 2 (0.8%) total deaths recorded in 2012, whose HIV status was not documented.*

Reviewer #2:

General comments to be considered once they revise the manuscript:
• Add continuous line numbers for easy commenting.

**Response:** *We have added continues line numbers in the revised manuscript.*

• Corresponding author name: why the name is all in small letters (abstract)?

**Response:** *We do not understand what the reviewer means, as the name of the corresponding author on the manuscript is not all in small letters.*

• Where applicable, use non-stigmatizing language like cases to TB patients.

**Response:** *The suggestion has been taken and applied.*

ABSTRACT
Background:
• ….” across a geographical location and to provide…” consider replacing the comma with and.

**Response:** *The correction has been made.*

Methods:
• Bivariate and multivariate logistic regression were used to identify predictors of TB deaths with Odds ratios and 95% confidence intervals estimated. What is the difference between bivariate and multivariate logistic regression? I would suggest you use simple and multiple logistic regression term.

**Response:** *We have considered replacing bivariate and multivariate with simple and multiple logistic regression in the revised manuscript (line 154).*

Results:
• no results of logistic regression analysis.

**Response:** *We provided results of logistic regression in the result section of the abstract (line 48-49).*

Conclusion
• how is high co-infection with HIV a proxy for week programme performance?
• TB case detection rate was low and remained stable, whereas co-infection with HIV and mortality rates were quite high, suggesting weak programme performance. Please consider revising the conclusion based on the findings. Comment on technical aspects of why TB notification is low and provide a normative guidance of how to improve TB notification.

**Response:** *We have revised the conclusion section of the abstract as recommended (line 50-53).*

MAIN ARTICLE
Background
• Worldwide, about 10 million incident cases of TB occur annually. Though correct, please consider mention the 10 million estimate corresponds to which year.

**Response:** *We have specified the year of the estimate in the revised manuscript (line 58).*

• Include estimates of death at global and WHO AFRO region, as mortality is one of the outcomes of interest.

**Response:** *We have included estimates of death at the global and WHO AFRO region in the revised manuscript (line 85-87).*

Methods
• Categorical variables were expressed in percentages and mean ± standard deviation (SD) for continuous variables, they were compared using Pearson’s chi-square test or Fisher’s exact test and the independent-samples t-test accordingly to describe the study population. The presentation of continuous variables into mean or median is dependent on their distribution. How did the authors make a decision to present directly means and SD? In your results, first paragraph, you have presented median and IQR.

**Response:** *We reported median and interquartile range because age was not normally distributed. We have replaced mean and standard deviation with median and interquartile range in the data analysis section of the revised manuscript as it was an error (line 51-52).*

• Case Detection Rate (CDR) was computed as the number of TB cases notified during the period divided by the estimated annual TB incidence per 100 000 Ghanaian population. Also, the reference is number 11 which is (World bank. The world bank data catalogue. <http://datacatalog.worldbank.org/publiclicenses#cc-by>), not sure if CDR definition and CDR data comes from world bank instead of WHO. The current term now used is the treatment coverage, do the authors care to explain their choice of using CDR instead of treatment coverage.

**Response:** *We used CDR for the sake of comparison since many published studies used CDR instead of treatment coverage. We have also replaced the world bank reference with that of WHO.*

• Data sources and extraction: change information to data and what are the TB/HIV collaborative activities data? TB/HIV collaborative activities do you limit yourselves to HIV testing and ART initiation? Or you also include IPT? Please be clear.

**Response:** *Information has been replaced with data. TB/HIV collaborative data included HIV status and ART treatment. We have made this clear in the revised manuscript (line 141). ART data in the registers were incomplete and there was no IPT data in the registers, hence were not included in our analysis.*

• Case definition: for pulmonary positive TB patients, are does the definition also include those with GeneXpert results?

**Response:** *GeneXpert were in use in some of the districts but were mainly used for testing suspected MDR-TB patients and not for routine testing, hence the definition did not include GeneXpert results.*

Results
• General results: presented median & IQR, but the not described this in methods section. The method section has only means and SD.

**Response:** *We have corrected the anomaly.*

• How did the author calculate estimated TB incidence for each district to come up with the CDR? Need to be clear in the methods section.

**Response:** *We used WHO’s estimated TB incidence in Ghana for each period under study and worked out the incidence for each district and each year using the yearly estimated district populations. For example, there were 148 incident TB cases per 100,000 Ghanaian population in 2019 [WHO 2019 TB report]. To estimate TB incidence for say district ‘A’ we used the formula; 148/100,000*the district population in a specific year.*

• Table 5: need to add a note, that the adjusted p-value is adjusted to what variables.

**Response:** *We adjusted for HIV status, type of TB and district of treatment. A note has been added to Table 5 to this effect as suggested (line 258).*

• Logistic regression analysis: have the authors considered the effect of inter-cluster correlations? Can the author justify removing sex in the regression analysis?

**Response:** *Since we used existing individual and not clustered data in our analysis, we did not consider the effect of inter-cluster correlations. Additionally, sex was included in the simple logistic regression analysis and since it did not show association with TB death, it was excluded from the multiple regression analyses.*

• From results: Similarly, smear-positive pulmonary TB patients were 66% (95% CI: 0.54-0.82; p<0.001) less likely to die during anti-TB treatment compared to smear-negative pulmonary TB patients. If the point estimates is in %, then the 95% CI should also be in %.

**Response:** *We have changed 66% to 0.66 odds ratio in the revised manuscript (line 251).*

Discussions
• Our analysis underscores significant district variation in the CDR and that underreporting of TB is a major problem in this part of the country, with an average of only 4 in 10 of the estimated cases being reported to the NTBCP. This statement is partly true, as under-reporting is one of the causes of such a discrepancy between incident and notified TB patients. However, under-diagnosis could also contribute to the gap. Are the authors confident that diagnostic capacity is optimal, and that the patients are diagnosed but not only reported. is this statement supported by an inventory study that documents under-reporting at least in those 10 districts?

**Response:** *We have indicated in the discussion section that* *the availability and proximity of diagnostic facilities and DOTS centres are likely to be an essential factor for case detection. This was based on the fact that districts with the lowest diagnostic facility density had the lowest CDR in this study. Even though this study did not assess diagnostic capacity of the districts, we compared our results with the diagnostic capacity of the districts studied as indicated in the regional NTP report and have referenced same.*

• Our finding falls short of the 70% target prescribed by the WHO for all national control programmes to reduce the number of people falling ill with TB by 90% and TB deaths by 95% by 2035. I am interested on how authors came to the conclusion that the country country falls short of reducing TB incidence and mortality by 2035. I don’t see any projections to back up their conclusion in this one.

**Response:** *We compared the 40% CDR estimated in our study with the WHO target of 90% and came a conclusion that our observed rate is lower than the WHO’s target. The statement did not imply that Ghana falls short of reducing TB incidence and mortality by 2035.*

• The deadly synergy between TB and HIV raises complexity and represent a considerable obstacle towards achieving global TB elimination target. The WHO, therefore, recommends systematic screening and documentation of HIV status among all TB patients enrolled on anti-TB treatment as an essential component of the TB care package [24], which is one of the core indicators for monitoring the implementation of the End TB strategy at all levels. The offer of HIV test for TB patients presents an opportunity essential for providing the best care for TB/HIV coinfected patients, including antiretroviral therapy [2]. Please consider deleting or shorten to a sentence or two. Most of the text is in the introduction part.

**Response:** *Most part of the text has been deleted from the revised manuscript.*

• Tuberculosis Case Fertility rate (CFR). Please change to fatality.

**Response:** *Fertility has been changed to fatality. That was a typographical mistake*

• From results: Similarly, smear-positive pulmonary TB patients were 66% (95% CI: 0.54-0.82; p<0.001) less likely to die during anti-TB treatment compared to smear-negative pulmonary TB patients. From discussion section: Additionally, we found that patients who were pulmonary smear-positive were 34% less likely to die during anti-TB treatment, compared to smear-negative pulmonary TB patients. I am bit confused with the presentation of these findings.

**Response:** *We have corrected the anomaly****.*** *Smear-positive patients were rather 0.66 times less likely to die during treatment compared to smear-negative patients.*

Thank you for considering this manuscript.

Sincerely yours,

**Signed**

Eric Osei

(Corresponding Author)
